# Uncovering Randomness and Success in Society

**DOI:** 10.1371/journal.pone.0088249

**Published:** 2014-02-12

**Authors:** Sarika Jalan, Camellia Sarkar, Anagha Madhusudanan, Sanjiv Kumar Dwivedi

**Affiliations:** 1 Complex Systems Lab, Indian Institute of Technology Indore, Indore, Madhya Pradesh, India; 2 Physics Department, Hindu College, University of Delhi, University Enclave, Delhi, India; Indian Institute of Technology Indore, India

## Abstract

An understanding of how individuals shape and impact the evolution of society is vastly limited due to the unavailability of large-scale reliable datasets that can simultaneously capture information regarding individual movements and social interactions. We believe that the popular Indian film industry, “Bollywood”, can provide a social network apt for such a study. Bollywood provides massive amounts of real, unbiased data that spans more than 100 years, and hence this network has been used as a model for the present paper. The nodes which maintain a moderate degree or widely cooperate with the other nodes of the network tend to be more fit (measured as the success of the node in the industry) in comparison to the other nodes. The analysis carried forth in the current work, using a conjoined framework of complex network theory and random matrix theory, aims to quantify the elements that determine the fitness of an individual node and the factors that contribute to the robustness of a network. The authors of this paper believe that the method of study used in the current paper can be extended to study various other industries and organizations.

## Introduction

The field of network analysis helps us to look at the study of an individual component as a part of a complex social structure and its interactions [1]. It explains various phenomena in a wide variety of disciplines ranging from physics to psychology to economics. The theory is adept at finding the causal relationships between network attributes such as the position of a node and the specific ties associated with it, and the fitness of the said node [2]. Such relationships, that seemed thoroughly random to the eyes of a researcher only about a decade before, have now been vastly studied and documented [3]. We aim to further investigate the very interesting idea that human behavior is predictable to a fair degree [4] using the Bollywood Network as a model for this purpose.

Making nearly one thousand feature films and fifteen hundred short films per year, the Indian film industry is the largest in the world [5] which has held a large global population in more spheres of its existence than just entertainment. It mirrors a changing society capturing its peaks and valleys over time and impacts the opinions and views of the diverse populace [6]. An example that can be stated as a proof of this was exhibited when the number of Indian tourists to Spain increased by 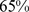 in the year succeeding the box office success of the movie ‘Zindagi Na Milegi Dobara’, which extensively portrayed tourist destinations in Spain, and also in the fact that Switzerland, depicted in various popular yesteryear Indian films (movies), remains a popular tourist destination for Indians to date [7].

The Hollywood co-actor network is a social network that has invited a fair amount of interest in the past [8], studies being conducted using relational dependency network analysis, Layered Label Propagation algorithm and PageRank algorithm [9], [10]. In comparison, its much larger counterpart in India has been largely ignored. Flourishing with a 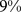 growth from 2009 to 2010 [7] and a further 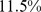 growth from 2010 to 2011 [11], it is an industry that sees blazingly fast growth, leading us to expect drastic changes in small time frames. We study the Bollywood industry because it provides a fair ground to capture the temporal changes in a network owing to its rapidly changing character. Using data from the past 100 years, we construct a network for every five year period. The nodes can be classified into the three distinct categories: 1) lead male actors, 2) lead female actors and 3) supporting actors. We analyze the structural properties of this network and further study its spectral properties using the random matrix theory (RMT).

Though originally rooted in nuclear physics [12], RMT has found widespread applications in different real systems such as the stock-market indices, atmosphere, human EEG, large relay networks, biological networks and various other model networks. Under the framework of RMT, such systems and networks follow the universal Gaussian orthogonal ensemble (GOE) statistics. Though there exist other universality classes such as Gaussian unitary ensemble and Gaussian symplectic ensemble [13], which have also been extensively investigated in RMT literature, we focus only on GOE statistics as spectra of various networks have been shown to rest with this universality class [14]–[16]. The universality means that universal spectral behaviors, such as statistics of nearest neighbor spacing distribution (NNSD) are not only confined to random matrices but get extended to other systems. A wide variety of complex systems fall under this class, i.e. their spectra follow GOE statistics ([17] and references therein).

## Materials and Methods

### Construction of Bollywood networks

We collect all Bollywood data primarily from the movie repository website www.bollywoodhungama.com and henceforth from www.imdb.com and www.fridayrelease.com (now renamed as www.bollywoodmdb.com) and we generate no additional data. The website www.bollywoodhungama.com previously known as www.Indiafm.com, is a reputed Bollywood entertainment website, owned by Hungama Digital Media Entertainment, which acquired Bollywood portal in 2000. We use Python code to extract names of all the movies and their corresponding information for a massive period of hundred years spanning from 1913 to 2012. Initially we document the names of all films as per their chronological sequence (latest to oldest) from the websites by incorporating the desired URL [18] in the code along with a built-in string function which takes the page numbers (932 pages in “Released before 2012” category and 24 pages in “Released in 2012” category) as input. Each film of every page bears a unique cast ID in the website, navigating to which via “Movie Info” provides us complete information about the film. In the Python code, we store the unique cast IDs of films in a temporary variable and retrieve relevant information using appropriate keywords from the respective html page. We also manually browse through other aforementioned websites in order to collect any yearwise missing data, if any. Thus we get the data in terms of names of the movies and names of the actors for 100 years. We then merge the data from all the websites and omit repetitions. A total of 8931 movies have been documented so far in Bollywood from 1913 till 2012. Harvesting the complete data took approximately 2000 hours of work over a 4-month period, which includes manual verification, formatting, removal of typos and compilation of the data. Considering the rapidly changing nature of the Bollywood network, we assort the curated massive Bollywood data in to 20 datasets each containing movie data for five-year window periods, as this is an apt time frame within which the network constructed is large enough to study the important network properties, and is not too large to miss any crucial evolutionary information. Since the number of movies and their actors in the time span 1913–1932 were scanty and could not have yielded any significant statistics, we merge the 1913–1932 datasets and present as a single dataset 1928–1932.

We create database of all actors who had appeared in the Bollywood film industry ever since its inception in five-year window periods, as mentioned in the previous version of the manuscript, by extracting them from the movie information using Python algorithm and we assign a unique ID number to each actor in every span which we preserve throughout our analysis. We take care of ambiguities in spellings of names of actors presented in different websites by extensive thorough manual search and cross-checking to avoid overlapping of information and duplication of node identities while constructing networks. Tracking by their unique ID numbers assigned by us, we create a co-actor database for each span where every pair of actors who had co-acted in a movie within those five years are documented. We then construct an adjacency list of all available combinations of co-actors. Treating every actor as a node and every co-actor association as a connection, we create a co-actor network of the largest connected component for every span.

We pick the actors appearing as the protagonist (occupant of the first position) in the movie star cast list from the movie star cast database created by us and observe that they incidentally are male actors in almost all movies with some rare exceptions. On extensive manual search based on popularity, award nominations we find that those male actors appear as a lead in the respective movies which made our attempt to extract lead male actors even easier. We could very well define the lead male actor as the protagonist in the star cast of at least five films in consecutive five-year spans and extract them from the movie star cast list using Python code while we were unable to find any proper definition for lead female actors as the second position of the movie star cast list is alternately occupied by either female actors or supporting actors, making it difficult to extract them only based on the network data as described. Hence we handpick the lead female actors from the movie star cast database for all the spans based on their popularity, award nominations and create their database.

### Assimilation of Filmfare awards data

We consider Filmfare award nominations as the best means to assess the success rates of all lead actors of Bollywood and distinguish the lead female actors from the rest. Filmfare awards were first introduced by the The Times Group [19] after the Central Board of Film Certification (CBFC) was founded by Indian central government in 1952 to secure the identity of Indian culture. The reason behind choosing Filmfare Awards amongst all other awards in our analysis is that it is voted both by the public and a committee of experts, thus gaining more acceptance over the years. Instead of the awards bagged we rather take into account the award nominations in order to avoid the interplay of some kind of bias affecting the decision of the CBFC committee in selecting the winner. By manual navigation through every year of Filmfare awards available on the web, we create a database of all categories of Filmfare awards and extract their respective nominees chronologically from the html pages using Python codes. Henceforth we use C++ codes to count the number of times every actor is nominated in each five-year span. Thus we obtain a complete list of all actors in each span along with their number of Filmfare nominations.

### Structural attributes of Bollywood networks

Considering 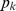 to be the fraction of vertices with the degree k, the degree distribution of the constructed networks is plotted with 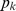. It has been sufficiently proven that the degree distribution of real world networks are not random, most of them having a long right tail corresponding to values that are far above the mean [1].

We define the betweenness centrality of a node i, as the fraction of shortest paths between node pairs that pass through the said node of interest [20].
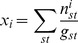
(1)where 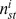 is the number of geodesic paths from 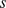 to 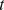 that passes through 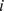 and 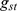 is the total number of geodesic paths from 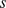 to 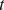.

### Measures used for success appraisal

In the current work, the concept of a payoff has been borrowed from the field of management [21], and adapted to suit the Bollywood network analysis. Payoff has elucidated the success of the center and non-center agents in a unique efficient star network [22]. We use an improvised version of payoff as a means to assess success rates of the nodes in Bollywood. For the purpose of devising net payoff (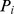), we study the datasets two at a time (accounting for ten years) and use the following definition:
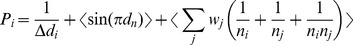
(2)where, 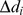 is the change in degree of a particular node 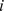 in two consecutive spans. 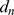 is its normalized degree in a particular span given as 
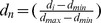
 with 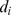 being the degree of the node i and 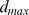 and 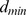 being the maximum and minimum degree in that particular span, respectively. The third term sums over all nodes j that node i has worked with where 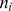 and 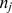 are the number of movies that the node i and j has worked in respectively and 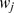 the number of times the node 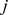 has worked with the node 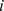 in the considered time window. The averages denoted in the net payoff (Eq. 2) refer to the values averaged over the two consecutive datasets. Based on the values of 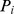, the actors of every set studied were ranked and lists made.

Due to the absence of a unifying framework that can be used to evaluate the success of films and their actors in the years before the inception of Filmfare Awards in 1954, we restrict our analysis on assessment of success to the time periods spanning from 1954 and onwards. In order to adumbrate the success of actors in the industry, we define overlap as the intersection of sets of co-actors that an actor has worked with, in two consecutive time frames.

### Spectral analyses

The random matrix studies of eigenvalue spectra consider two properties: (1) global properties such as spectral distribution of eigenvalues 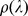, and (2) local properties such as eigenvalue fluctuations around 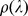. Eigenvalue fluctuations is the most popular one in RMT and is generally obtained from the NNSD of eigenvalues. We denote the eigenvalues of a network by 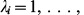 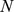 and 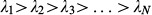. In order to get universal properties of the fluctuations of eigenvalues, it is customary in RMT to unfold the eigenvalues by a transformation 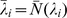, where 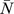 is average integrated eigenvalue density. Since we do not have any analytical form for 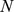, we numerically unfold the spectrum by polynomial curve fitting [12]. After unfolding, average spacings are unity, independent of the system. Using the unfolded spectra, spacings are calculated as 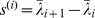.

The NNSD is given by
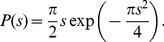
(3)For intermediate cases, the spacing distribution is described by Brody distribution as
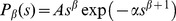
(4)where 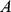 and 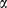 are determined by the parameter 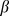 as follows:
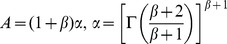
This is a semi-empirical formula characterized by parameter 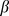. As 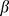 goes from 0 to 1, the Brody distribution smoothly changes from Poisson to GOE. Fitting spacing distributions of different networks with the Brody distribution 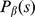 gives an estimation of 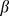, and consequently identifies whether the spacing distribution of a given network is Poisson, GOE, or the intermediate of the two [23].

The NNSD accounts for the short range correlations in the eigenvalues. We probe for the long range correlations in eigenvalues using 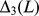 statistics which measures the least-square deviation of the spectral staircase function representing average integrated eigenvalue density 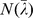 from the best fitted straight line for a finite interval of length 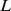 of the spectrum and is given by
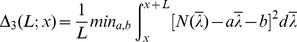
(5)where 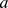 and 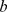 are regression coefficients obtained after least square fit. Average over several choices of x gives the spectral rigidity, the 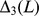. In case of GOE statistics, the 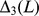 depends logarithmically on L, i.e.
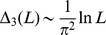
(6)


## Results and Discussion

### Structural properties of Bollywood networks

The degree distribution of the Bollywood networks follow power law, as expected based on the studies of other real world networks [1]. But an observation that defies intuition is that the most important nodes of the industry, acknowledged as the lead male actors, do not form the hubs of the constructed network, but instead have a moderate degree and also maintain it along sets of data that were studied (Tables S2–S7 in File S1). Considering the network on an evolutionary scale, this is a property that gains more prominence during the later sets of the data, while the network maintains power law throughout the entire timespan (Figure S1 in File S1). The prominent supporting actors of the era form the hubs of the industry in respective time frames. This counterintuitive nature of the above observation can be explained by the fact that these actors collaborate with more nodes and take on more projects in a given time period. Hence they can be said to be instrumental in establishing connections in the network. The scale-free behavior of the Bollywood industry can be elucidated by the fact that newcomers in the industry in general aspire to act with the lead actors of the era, who intuitively form associations with high degree nodes, thus illustrating the preferential attachment property prevalent in Bollywood networks [1].

### Success appraisal of Bollywood actors

By virtue of the sinusoidal function used in (Eq. 2), the nodes with a moderate degree lead the net payoff list with both low degree and high degree nodes trailing behind. The inverse of the change in degree favors nodes that preserve their degree over the years hence giving a higher net-payoff to actors who preserve their degrees over the various datasets.

**Figure 1:**
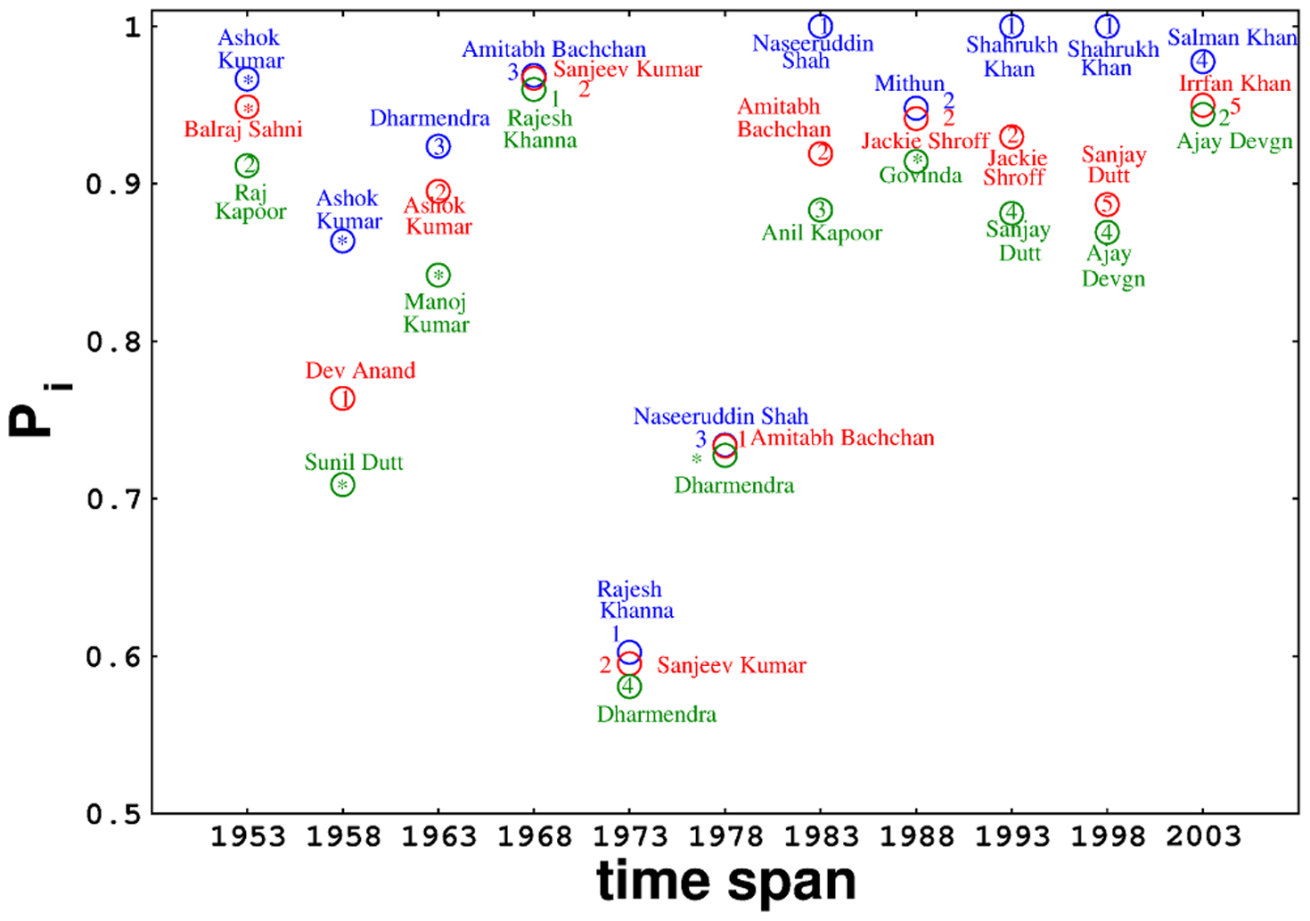
Net payoff (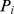) of top three lead male actors in each time span plotted against the respective time frames. They are ranked (as 1, 2 and so on) based on their number of Filmfare award nominations. ‘*’ denotes no Filmfare award nominations. Actors and their corresponding rankings are represented in same color.

**Figure 2:**
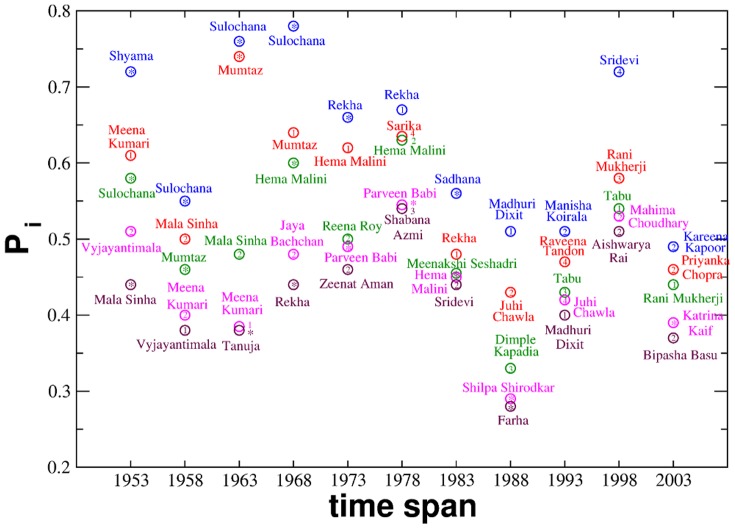
Net payoff (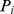) of top five lead female actors in each time span plotted against the respective time frames. They are ranked (as 1, 2 and so on) based on their number of Filmfare award nominations. ‘*’ denotes no Filmfare award nominations. Actors and their corresponding rankings are represented in same color.

Successful supporting actors, although bear a high degree, appear quite high in the scale of 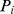 because they have relatively higher values of 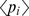. Though interplay of various contrasting factors influence the appearance of lead male actors in 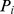 list, they appear high in absolute scale of 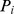 in all the sets under consideration except the ones corresponding to 1973–77 and 1978–82. Three of the top five Filmfare award nominees in lead male actor category appear as top three lead male actors in 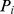 list in respective time frames (Figure 1 and Tables S2–S7 in File S1). This observation is more pronounced in case of the lead female actors. As observed in Figure 2 and Tables S8–S13 in File S1, the three lead female actors having secured the maximum number of Filmfare award nominations in a particular span of time, appear as the leading nodes in their respective 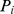 list, a trait that is more consistent in the more recent datasets. From the above analysis based on payoff it is supposed that possessing moderate degree and maintaining it are properties followed by the nodes that stand successful in Bollywood industry and can be contemplated as keys to success.

**Figure 3:**
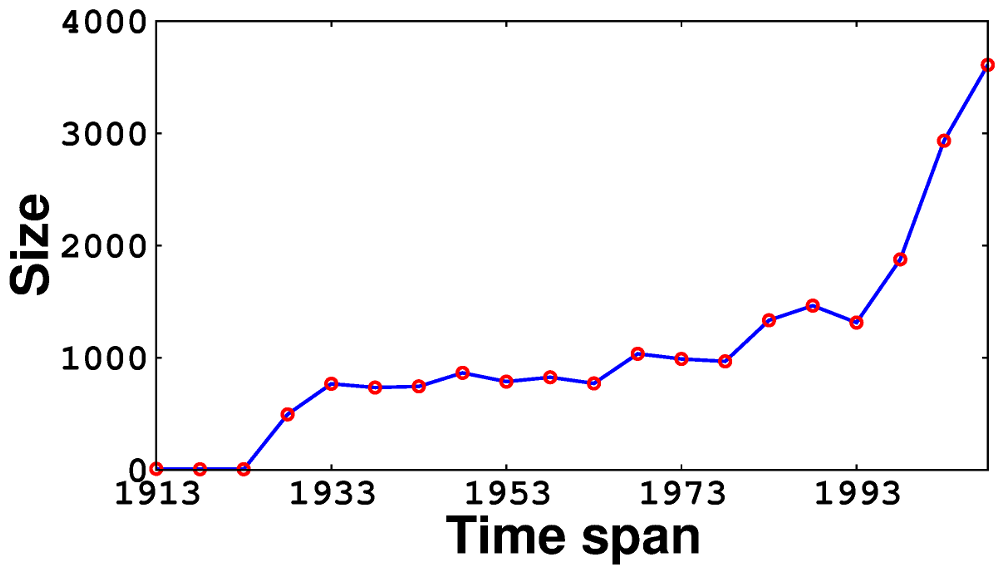
Evolution of Bollywood network size over 1913–2012.

Succeeding the economic liberalization in 1991, the inclusion of diverse socio-political-economic issues in mainstream Bollywood movies found favor with the audience [24]. At around this period, Hollywood started gaining popularity among the Indian population owing to the advent of private movie channels and the internet. These factors coupled together affected the structure of the network, which might be the underlying reason behind the observed variations in the network properties, pre, post and during liberalization. A steep rise in the Bollywood network size 1993 onwards (Figure 3) might be one of the manifestations of this shift in economic policies. The status of an ‘industry’ being conferred upon Bollywood in 1998 might be a result of this increased size of the network [25]. The comparatively larger shift of the network properties with the advent of liberalization as opposed to that caused by the introduction of the Filmfare awards in 1954, can lead us to conclude that mainstream Bollywood is largely driven by economic concerns rather than artistic ones.

**Figure 4:**
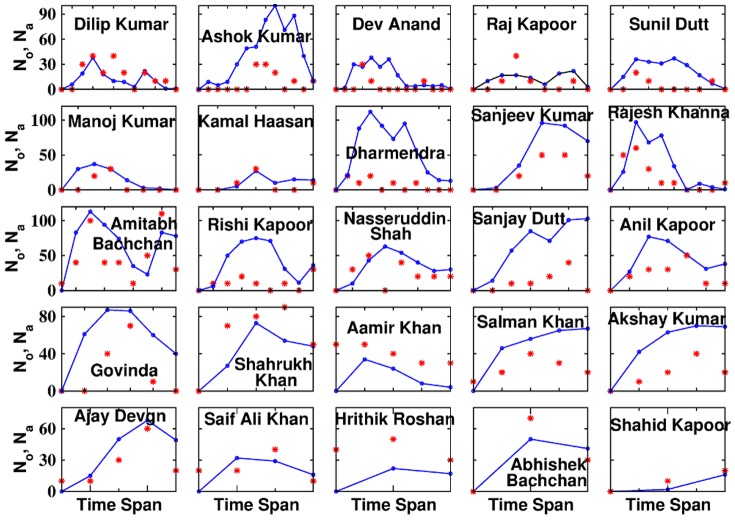
Plots of individual overlaps 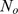 (represented by 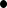) of lead male actors and their Filmfare award nominations 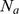 (represented by 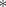) against their respective time spans. Time span here represents respective individual spans of lead male actors in Bollywood industry, for example Dilip Kumar had a long span stretching between 1943 and 1998 whereas Hrithik Roshan has a short spell 1998 onwards.

The number of times an actor is nominated for the Filmfare awards while they remain a lead actor, when plotted with their overlap (as defined before), shows that 22 among the 25 actors exhibit an approximate direct proportionality (Figure 4) emphasizing on the importance of winning combinations. Overlap being one of the probable factors deciding the success of a node might explain the reason for the formation of social groups, and co-operation among them in the society [26].

**Figure 5:**
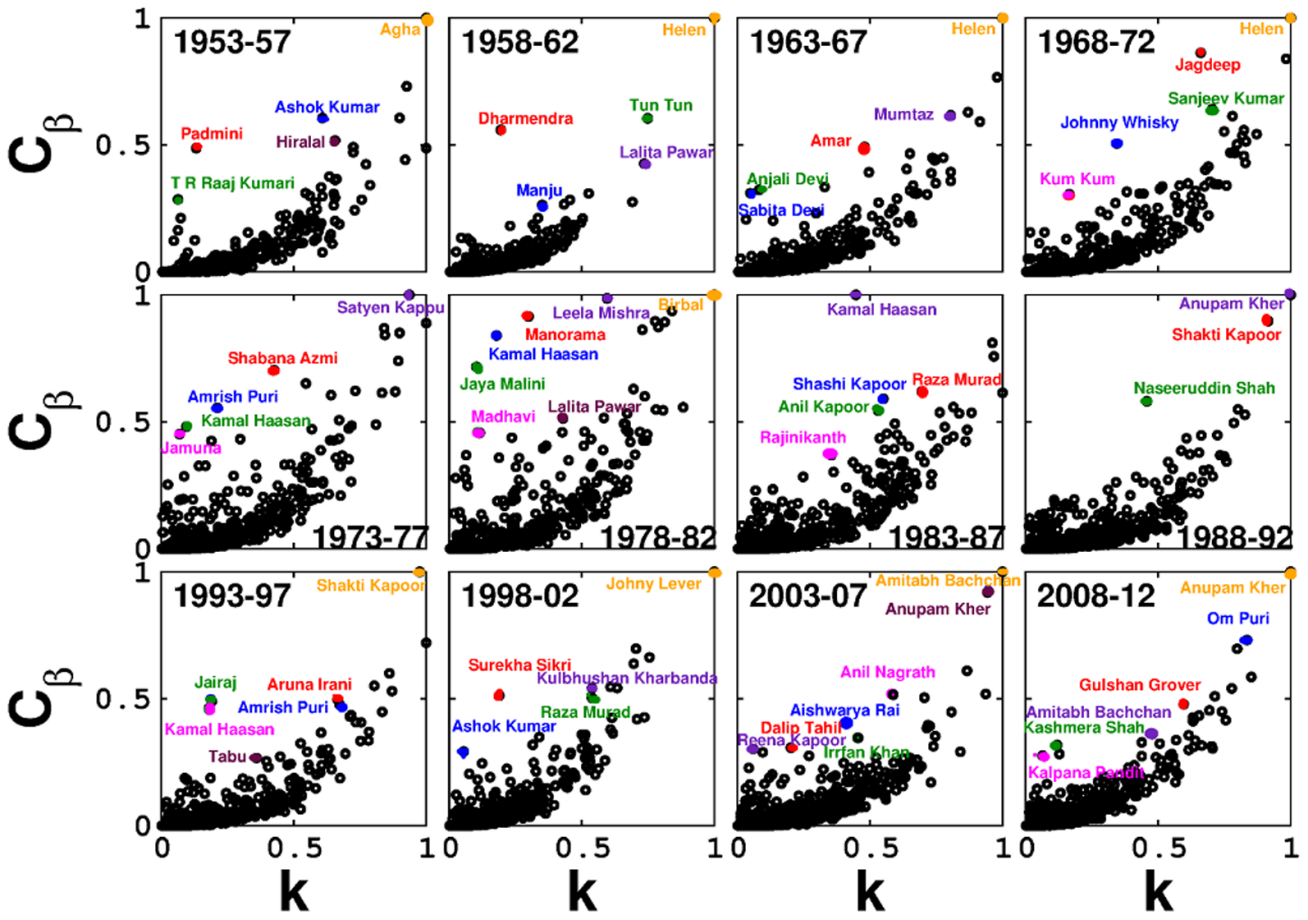
Plots of normalized betweenness centrality (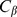) against normalized degrees (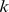) of Bollywood actors over 1953–2012. Actors and their corresponding betweenness centrality are represented in same color.

High degree nodes indubitably have high betweenness centrality. Actors with high betweenness centrality seem to have a relatively larger span in the industry even if their popularity levels, measured as the number of Filmfare award nominations, is not markedly high. Nodes with the highest betweenness centrality of all datasets are found to be male actors (except Helen), whether lead or supporting, adumbrating the gender disparity in Bollywood. Incidentally, few of the nodes bearing moderate and low degree also exhibit high betweenness centrality and also have a long span in the Bollywood industry (Figure 5; Figure S2 and Table S1 in File S1). This indicates that actors exhibiting mobility between diverse Bollywood circles seem to have an advantage of a long span, though we are far from concluding that this is the only factor affecting the life span of a node. There exist examples from social and biological systems which also support the importance of cooperation and mobility [27].

### Spectral analyses of Bollywood networks

The spectral density, 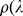) of the connectivity matrix of Bollywood networks exhibit a triangular distribution (Figure S3 and discussion in File S1), hence providing evidence supporting its scale-free nature [28]. The eigenvalue distribution of the Bollywood networks show a high degeneracy at 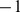, deviating from the commonly observed degeneracy at 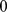 in most of the real world networks studied (for example, biological networks [14]). This degeneracy at 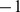 can be attributed to the presence of clique structures in the network [29]. Presence of dead-end vertices in spectrum and motif joining or duplication have been used as plausible explanations to widespread degeneracy at 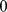 observed in biological networks [30].

**Figure 6:**
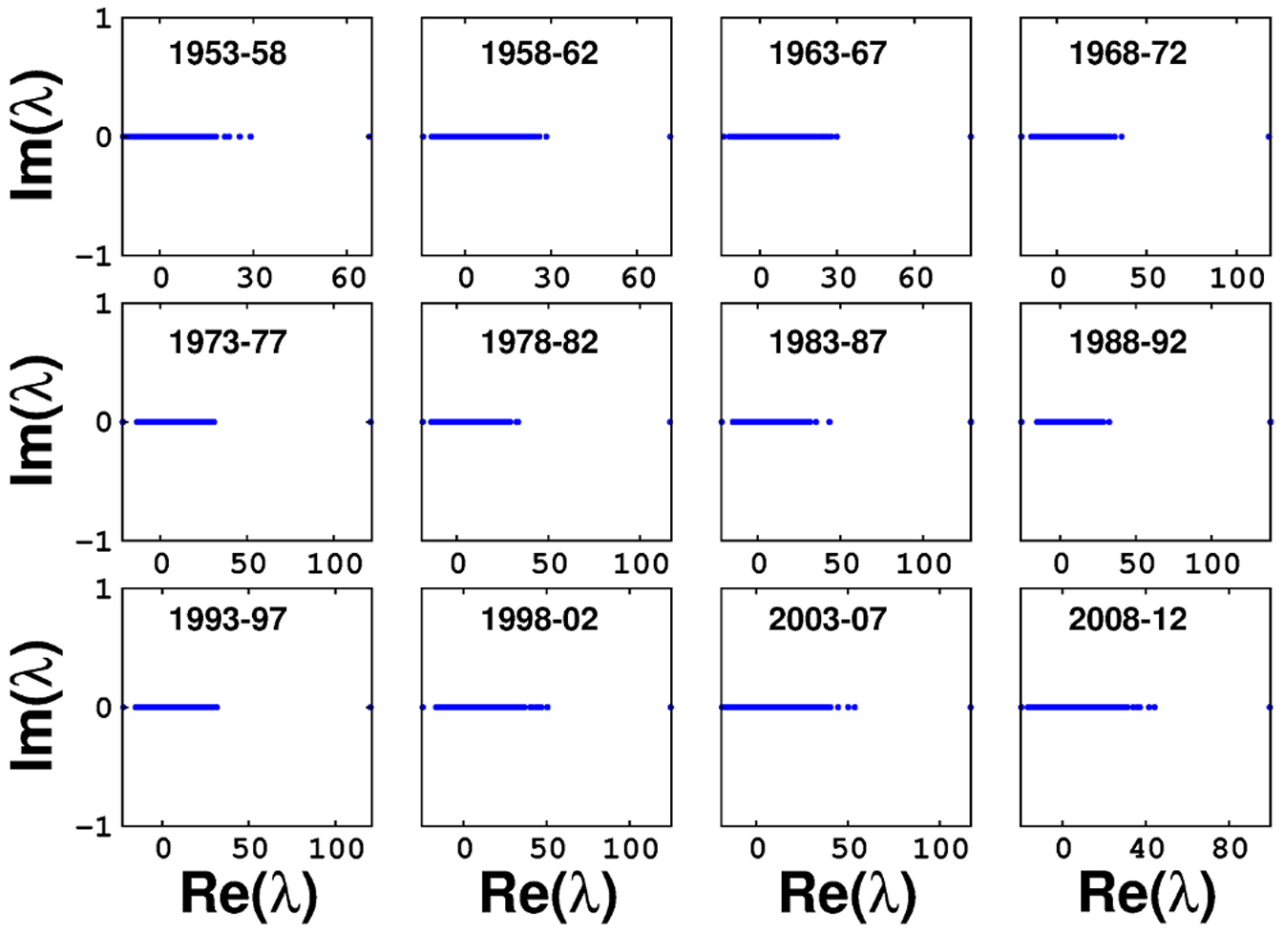
Separation of lone eigenvalues from bulk of eigenvalues in Bollywood datasets spanning between 1953–2012.

Factors affecting a social network are vastly different from those affecting a biological network, hence making the nature of their spectra varied. Owing to a relatively smaller number of nodes in the networks constructed for the periods 1913–17, 1918–22 and 1923–27, a bulk does not appear in their eigenvalue distributions. The distributions corresponding to the datasets of 1928–57, 1983–87 and 2003–12 very clearly show the presence of a few eigenvalues outside the bulk (Figure S4 in File S1 and Figure 6), which is formed by the rest of the eigenvalues. While the largest eigenvalue is distinctly separated from the bulk, which is a well-known spectral feature of an undirected network [20], existence of other eigenvalues outside the bulk probably indicate the existence of distinct Bollywood guilds [31] further portending an evolving network structure.

The spectral data as well as the data regarding the betweenness centrality of the networks, corresponding to the time periods after 1998–02, suggest that there has been a drastic change in the underlying network structure since then. This marked change in the more recent datasets in comparison to the older ones, is clearly illustrated by the presence of several eigenvalues outside the bulk (Figure 6), and the presence of a lesser number of low degree nodes with a high betweenness centrality (Figure 5). This indicates that the community structures in the Bollywood network have gotten more inter-interconnected post 1998–02, leading the authors of this paper to conclude that Bollywood is becoming increasingly systematic with time.

We fit the NNSD of Bollywood networks by the Brody distribution (Eq. 4) and find that the value of 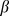 comes out to be close to 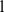 for all the datasets. This implies that the NNSD of Bollywood datasets follow GOE statistics of RMT (Eq. 3 and Figure S5 in File S1) bringing Bollywood networks under the universality class of RMT [15], [17]. To examine the long range correlations, we calculate spectral rigidity via the 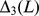 statistics of RMT using Eq. 5 by taking same unfolded eigenvalues of different datasets as used for the NNSD calculations. The value of 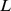 for which the 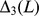 statistics follows RMT prediction (Eq. 6) is given in the Table 1 and the detailed plots are deferred to File S1 as Figure S6. The 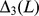 statistics which provides a measure of randomness in networks [16] clearly indicate that the dataset corresponding to the 1963–67 timespan has the most random underlying network structure when compared with the other datasets. This notable feature of this timespan can probably be attributed to the consecutive wars that India was a part of in the years 1962 and 1965, which in turn lead to an extreme economic crisis in the country. As shown by the decreasing value of L since 1933, the networks have a trend of diminishing randomness.The dataset corresponding to 1948–52 witnessed a breach from this trend, probably due to the drastic political and financial changes post Indian Independence in 1947. One of the most crucial points exhibited in the analysis based on eigenvalue distribution and betweenness centrality is that, before the year 1998 the structure of the networks had either well segregated clusters or extreme random interactions, while post 1998 the structures seem to maintain a fairly consistent randomness (randomness measured by the value of L).

**Table 1:** Properties of Bollywood network of each 5 years block datasets.

Time span	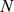	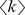	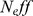	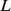	% 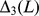
1928–32	496	9.46	162	8	4.93
1933–37	769	10.7	246	6	2.43
1938–42	735	13.3	248	5	2.02
1943–47	745	12.6	276	5	1.81
1948–52	866	17.5	291	8	2.75
1953–57	788	25.9	272	-	-
1958–62	827	29.9	313	-	-
1963–67	772	35.2	308	19	6.16
1968–72	1036	47.0	416	-	-
1973–77	990	47.5	383	14	3.65
1978–82	968	45.1	370	16	4.32
1983–87	1335	44.6	480	19	3.95
1988–92	1465	44.9	546	24	4.39
1993–97	1314	42.2	504	12	2.38
1998–02	1878	46.3	686	14	2.04
2003–07	2935	37.0	973	17	1.74
2008–12	3611	30.3	1164	17	1.46

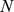
 and 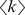 respectively denote size and average degree of network. 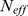 and 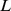 are the effective dimension of non-degenerate eigenvalues less than 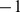 and the length of the spectrum up to which spectra follow RMT. % The 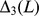 represents the extent of 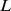 2 which spectra follow GOE statistics, expressed in percentage terms. ‘-’ denotes the spectra which do not follow RMT.

## Conclusions

Although Bollywood networks for different spans demonstrate varying amounts of randomness as suggested by the changing values of L in the 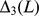 statistics, observation of universal GOE statistics of the NNSD puts forward the evidence to show that a sufficient amount of randomness is possessed by all the sets. The efficiency of many real world systems such as the financial markets, the climatic system, neuronal systems etc, has been aided by their stochastic nature which leads to randomness [32]. Bollywood network also provides an example to aid this relationship, as the industry has survived various valleys and crests since its inception, including in times of dire socio-economic crisis [33].

The extensive analyses of Bollywood data on the one hand reveals its influence on the decisions and preferences of the mass, while on the other it unravels the prevailing gender disparity [34], [35] thus acting as a reflection of the society. Furthermore, it helps us deduce that cooperation among the nodes leads to combinations that become formulaic for successful ventures. It also seems to further propagate the idea suggesting that a combination of organization and randomness in the network structure supports the sustenance of the represented network. We believe that the analysis of the Bollywood network as carried out in this work can be extrapolated to study the predictability of success and the ingredients that are necessary for the robustness of other social collaboration networks [36] and organizations [37].

## Supporting Information

File S1
**Supporting information file for the article “Uncovering randomness and success in society”.** It contains the plots of betweenness centrality and eigenvalues for 1928–1952 datasets, plots of degree distribution, nearest neighbor spacing distribution and 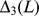 statistics and list of actors who have high betweenness centrality (alongwith their span in Bollywood and recognition) for 1928–2012, plot of spectral density distribution and lists of top 10 lead male and top 5 female actors based on their net payoff alongwith their award nominations for 1953–2012 datasets.(PDF)
