# Supplementary material for: Uncovering Randomness and Success in Society
Source: PLoS One. 2014 Feb 12;9(2):e88249. doi: 10.1371/journal.pone.0088249 (PMC3922759; doi:10.1371/journal.pone.0088249)
Supplement: File S1 — Supporting information file for the article “Uncovering randomness and success in society”. It contains the plots of betweenness centrality and eigenvalues for 1928–1952 datasets, plots of degree distribution, nearest neighbor spacing distribution and statistics and list of actors who have high betweenness centrality (alongwith their span in Bollywood and recognition) for 1928–2012, plot of spectral density distribution and lists of top 10 lead male and top 5 female actors based on their net payoff alongwith their award nominations for 1953–2012 datasets. (PDF) [file pone.0088249.s001.pdf]

Supporting Information

Uncovering randomness and success in society

Sarika Jalan, Camellia Sarkar, Anagha Madhusudanan, Sanjiv Kumar Dwivedi

1 Methods

Study of society and its movement has traditionally involved obtaining data from representative populations through field studies and extrapolating the obtained results through approximations [1]. These methods of data collection provide, in the first place incomplete data and secondly, data that is prone to errors that would drastically skew the results obtained by the physicists' method of studying them. Movie actors networks analyses became a lucrative means for assessing society as the data obtained is to a satisfiable extent accurate and free from approximations and bias.

Although individual endowments (income) should rationally be the apt discriminating factor for distinguishing lead actors from the supporting ones, it is quite cumbersome to retrieve relevant data due to lack of reliable sources meant for the same. The variable nature of the data adds to its impediment. We define lead male actors based on the number of times they top the starcast list in consecutive spans while defining lead female actors still remains an agony even after a century of cinematic heritage (discussed in sufficient detail in the main article). Although movies like Fashion, Page 3, Chandni Baar, Kahaani, Heroine portrays the never ending struggle of women in society, the basis of their struggles have undoubtedly changed over the years. While Mother India (1957) depicts the struggle for existence, a struggle to combat poverty, Fashion (2008) depicts a struggle for fame, a struggle for passion, a struggle for touching dreams, but

not a struggle for existence. This reflects a gradual change in the outlook of the society towards women.

In order to assess success of all actors in Bollywood industry, the Filmfare Awards were introduced for rewarding both artistic and technical excellence of professionals in the Hindi language film industry of India. The National Film Awards were also introduced in 1954 but gained less popularity as compared to Filmfare as they are decided by a panel appointed by Indian Government and do not authentically reflect the choice of the global audience. The Filmfare Awards, in contrast, are voted for by both the public and a committee of experts thus gaining more acceptance over the years.

1.1 A brief review of Hollywood networks

The collaboration graph of film actors were shown to be small-world networks [2] and their properties were studied using random graph theory [3]. Relational dependency network analysis has been performed on Hollywood datasets obtained from IMDB which identify and exploit cyclic relational dependencies to achieve significant performance gains [4]. Hollywood datasets were deployed for implementation of the Layered Label Propagation algorithm, meant to reorder very large graphs [5] and the PageRank algorithm to uncover the relative importance of a node in a graph [6]. Professional links between movie actors was used as a means to fit the predictions of a continuum theory to probe for the existence of two regimes, the scale-free and the exponential regime [7].

1.2 Structural Analyses

1.2.1 Degree Distribution

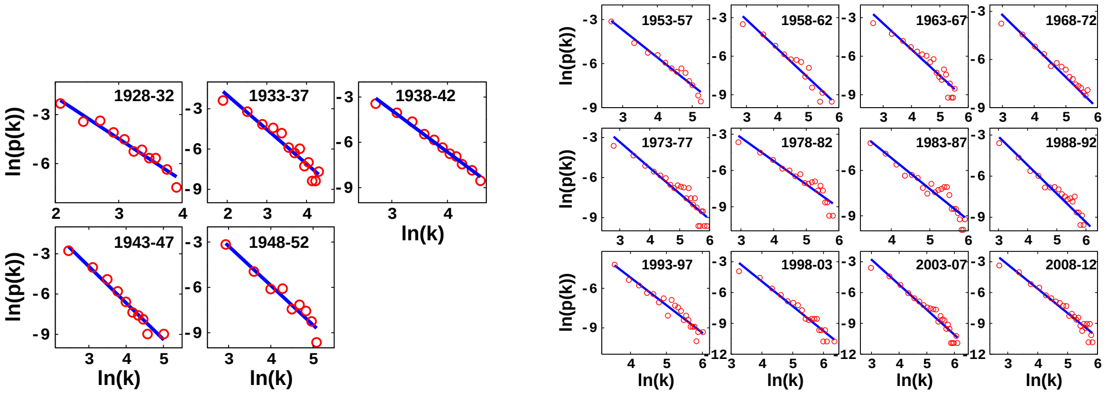

Figure S1: Degree distribution of the Bollywood networks over 1913-2012. Due to scarcity of actors in 1913-1927, all nodes appearing in 1913-27 have been merged and included in 1928-32.

Degree of a node can be defined as the number of nodes that are linked to the said node. Degree distribution is the plot of the degree versus the number of nodes with the particular degree. Fig.S1 plots degree distribution of Bollywood networks.

1.2.2 Betweenness Centrality

The supporting actors have been observed to have high betweenness centrality. Nodes having higher degree would naturally be coming into shortest path between pair of nodes, and hence would have high betweenness centrality. Fig.4 of main article and Fig.S2 has highest C_β corresponding to node possessing highest degree. The fact that larger degree in any of the sets in 1928-2012 are possessed by supporting actors, and it is somewhat established that supporting actors have longer life span than lead male actor and lead female actors, makes the positive correlation between degree and life span quite obvious. But some of the low degree nodes are also seen to have high betweenness centrality. Either they are supporting actors which again comply with the earlier argument for their larger life span, or if they are lead male actors then

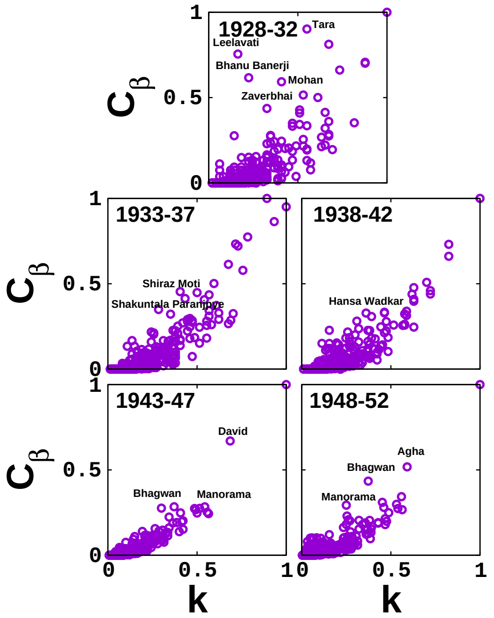

Figure S2: Plots of normalized betweenness centrality (C_β) against normalized degrees (k) of Bollywood actors over 1913-1952.

also they show accredited life span. For example, in 1958-62 dataset, Dharmendra having low degree distinctly appears in the high betweenness centrality region and has a remarkably long span (1953-2012) in the industry. Few other prominent actors who have been seen to follow this trend are Kamal Haasan (1958-2012), Nasseruddin Shah (1973-2012), Rajinikanth (1973-2012), Anil Kapoor (1978-2012). These examples are taken for those who are clearly depicting high betweenness centrality than rest of the nodes around them. Various female actors having low degree also fall in high betweenness centrality region and have long span. Padmini (1948-77) and Rajinikanth (1973-2012) are Tamil actors who have been observed in high betweenness centrality region bridging the gap between communities of Bollywood and Kollywood (Table S1).

Table S1: List of prominent actors who appear high in betweenness centrality zone

Names of actors	Span	Recognition
Agha	1937-1989	Known for comic roles, won Filmfare Best Supporting Actor Award (1960)
Ashok Kumar	1936-1993	An iconic figure in Indian cinema popularly known as “Dadamoni” who is also a painter, homeopath, astrologer, boxer, chess player, singer ; confered with honors like Dadasaheb Phalke award (1988) and Padma Bhushan (1998), Filmfare Lifetime Achievement Award (1995), Sangeet Natak Akademi Award (1959), National Film Awards for Best Actor (1969), Filmfare awards (1962, 1966, 1969)
Padmini	1948-1994	An elegant **Tamil** dancer who was also featured in several Hindi films; won Filmfare Award for Best Supporting Actress (1966)
Hiralal	1928-1995	A prominent supporting actor having a long span in industry
T R Rajakumari	1936-1955	Originally a Tamil film actress, Carnatic singer and dancer also acted in many Bollywood films
Helen	1951-2012	An Indian film actress and one of the most popular dancers of all times; has bagged Padma Shri (2009), Filmfare Best Supporting Actress Award (1979), Filmfare Lifetime Achievement Award (1998)
Tun Tun	1946-1990	A highly rated playback singer who later became a permanent comic relief in numerous Bollywood films.
Dharmendra	1960-2012	Often referred to as the “He-Man”, he has won Padma Bhushan (2012), Filmfare Lifetime Achievement award (1997), Filmfare Best Actor awards (1967, 1972, 1974, 1975), the Living Legend award (FICCI) and many more
Lalita Pawar	1928-1997	Known for her roles as wicked matriarch and mother-in-law, she has won Filmfare Best Supporting Actress Award (1959) and Sangeet Natak Akademi Award (1961)
Mumtaz	1952-1976	Critically acclaimed highly paid actress who has bagged a Filmfare Award for Best Actress (1970) and Filmfare Lifetime Achievement Award (1996)
Anjali Devi	1936-1994	A veteran Telugu and Tamil actress well known for her mythological roles in Bollywood
Sabita Devi	1924-1996	Supporting female actor
		continued

Table S1 — continued

Jagdeep	1951-2012	Especially known for his excellent comic timing and appearances in horror movies and character roles.
Sanjeev Kumar	1960-1985	An accomplished Indian film actor remembered for his versatility and genuine portrayals of characters; has bagged National Film Award for Best Actor (1971, 1973), Filmfare Award for Best Actor (1976, 1977)
Johnny Whisky	1961-1997	Popular supporting male actor
Kum Kum	1954-1973	With her sumptuous dancing talent, she has starred with superstars of the era
Satyen Kappu	1952-2007	A remembered character actor of Bollywood films
Shabana Azmi	1974-2013	Regarded as one of the finest Indian actress of film, television and theatre proficient in a variety of genres with a record of five wins of the National Film Award for Best Actress (1975, 1983, 1984, 1985, 1999), Filmfare Best Actress award (1978, 1984, 1985), Filmfare Lifetime Achievement award (2006) and several international honours
Amrish Puri	1954-2005	Primarily remembered for essaying iconic negative roles in Bollywood and international film industries; has Filmfare Best Supporting Actor awards (1986, 1997, 1998), Sangeet Natak Akademi Award (1979)
Kamal Haasan	1959-2013	Critically acclaimed Indian film actor, screenwriter, producer, director, songwriter, playback singer and choreographer; has won a record 19 Filmfare Awards ranging across five languages, four National Film Awards, Padma Shri, one Rashtrapati Award for Best Child Artist and several other state, national and international honours.
Jamuna	1954-1968	A veteran Telugu actress who has also won Filmfare Best Supporting Actress award (1968) for a Hindi movie.
Birbal	1966-2011	A veteran comedian who has acted in 377 Bollywood films.
Leela Mishra	1936-1986	A character actress with roles varying from mothers, benign or evil aunt to comic roles; has acted in over 200 Hindi films
Manorama	1941-2005	A Bollywood character actress, acted in over 160 films, known best for her role as the comical tyrant mother or villainous roles
Jaya Malini	1976-1988	Has acted in over five different languages; known for her dance and vamp roles
		continued

Table S1 — continued

Madhavi	1981-1994	Indian film actress acted in 7 languages in about 300 films
Raza Murad	1965-2013	With a rich baritone voice, he often portrays negative character roles
Shashi Kapoor	1941-1999	An award-winning Indian film actor, director and producer-Padma Bhushan
Anil Kapoor	1980-2013	One of the most successful actors of Bollywood with National Film Award for Best Actor (2001), Feature Film (2008), Filmfare Best Actor Award (1989, 1993, 98), Filmfare Best Supporting Actor Award (1985, 2000)
Rajinikanth	1975-2013	Being one of the highest paid actors of Asia, he is a cultural icon holding a matinee idol status; has been bestowed Padma Bhushan (2000)
Anupam Kher	1982-2013	A versatile Indian actor who has appeared in nearly 450 films and 100 plays in almost all possible genres including international Oscar nominated films; honoured with Padma Shri (2004), National Film awards (1989, 2005), Filmfare awards (1984, 1988, 1989, 1990, 1991, 1992, 1993, 1995)
Shakti Kapoor	1978-2012	One of the leading villains in Bollywood movies also applauded for his comic roles; bagged Filmfare Best Comedian Award (1995)
Naseeruddin Shah	1972-2013	Considered to be one of the finest Indian stage and film actors; recipient of Padma Shri (1987), Padma Bhushan (2003), National Film awards (1979, 1984, 2006), Filmfare awards (1981, 1982, 1984, 1993, 1995, 1996, 1998, 2000, 2007, 2008), Best Actor Venice Film Festival (1984)
Aruna Irani	1961-2010	A popular supporting actress, has acted in over 300 films Filmfare Best Supporting Actress Award (1985, 1993), Filmfare Lifetime Achievement Award (2012)
Jairaj	1929-1995	A renowned film actor, director and producer; recipient of Dadasaheb Phalke Award for lifetime achievement (1980)
Tabu	1980-2013	Garnered critical appreciation for acting in artistic, low-budget films across five languages; won Padma Shri (2011), National Film Award for Best Actress (1997, 2002), Filmfare awards (1995, 1998, 2000, 2001, 2007)
Johny Lever	1984-2013	One of the most popular comedians in Hindi cinema, has won Filmfare Best Comedian Award (1998, 1999) including 13 nominations,
		continued

Table S1 — continued

Kulbhushan Kharbanda	1974-2013	A popular Indian film, television actor, has been portrayed in a variety of roles ranging from a bald villian, doctor, police, hero to character roles; nominated for Filmfare Best Supporting Actor Award (1986)
Surekha Sikri	1978-2006	An Indian film, theatre and TV actress recently popular as the negative diva of telly wood, has won National Film Award for Best Supporting Actress (1988, 1995), Sangeet Natak Akademi Award (1989)
Anil Nagrath	1966-2013	Popular supporting actor
Aishwarya Rai	1997-2013	Winner of Miss India and Miss World pageants (1994) is a leading contemporary actress of Indian cinema proficient in a range of genres; Padma Shri (2009), Filmfare Best Actress Award (1999, 2002), Most Glamorous Star of the Year (2007), Outstanding Achievement in International Cinema (2009), Decade of Global Achievement Honour (FICCI, 2011)
Dalip Tahil	1974-2012	Indian film, television and theatre actor known primarily for his negative roles has also demonstrated his versatality playing character roles in a series of national and international television serials and films
Irrfan Khan	1988-2013	India's best known international actor skilled in performing in a variety of genres; has Padma Shri (2011), Filmfare Awards (2003, 2007, 2012), Screen Actors Guild Award (2008), IRDS Film Award for social concern (2012) to his credit
Gulshan Grover	1980-2013	An Indian actor and film producer known for his villainous roles and later for his comic roles as well; has many national and international honours to his credit
Kashmera Shah	1994-2011	An Indian actress and model who has won beauty contests
		continued

Table S1 — continued

Om Puri	1976-2013	Critically acclaimed for his performances in many unconventional roles in both mainstream Indian films and art films; winner of Padmashri (1990), National Film Award for Best Actor (1982, 1984), Filmfare awards (1981, 2009), Karlovy Vary International Film Festival Best Actor (1984), Brussels International Film Festival Best Actor (1998), Grand Prix Special des Amriques Montral World Film Festival for cinematographic art (1998)
Kalpana Pandit	2000-2013	An emergency physician, who turned into an Indian film actress and model; has hosted technical awards ceremony and has made red carpet appearances at Hollywood premier nights
Reena Kapoor	2000-2013	An Indian actress in films and television serials.

1.3 Spectral Analyses

Paul Erdös and Alfred Rényi pioneered the study of random graph models [10], which persisted as a preferred method for studying networks for decades. Following this, the Barabási-Albert model of networks suggested that many complex networks follow a power law degree distribution, hence forming what is termed as scale free network, which emerged as a revolutionizing change in network analysis and completely changed the perspectives of the analysts [11]. Some of the popular networks studied henceforth namely the Internet, the World-Wide-Web, cellular networks, phone call networks, science collaboration networks etc. appeared to follow the power law distribution [8]. For the undirected networks constructed here all the eigenvalues are real. We observe a high degeneracy at $\lambda = -1$, with almost 40% of states having this value. The presence of degeneracy at -1 is attributed to abundance of clique structure in underlying network probably arising due to several actors appearing in a same movie. Eigenvalue statistics of Bollywood network elucidate typical triangular structure, as observed for scale free networks

[12, 13], with a crucial difference in having peak at -1 (Fig. S3).

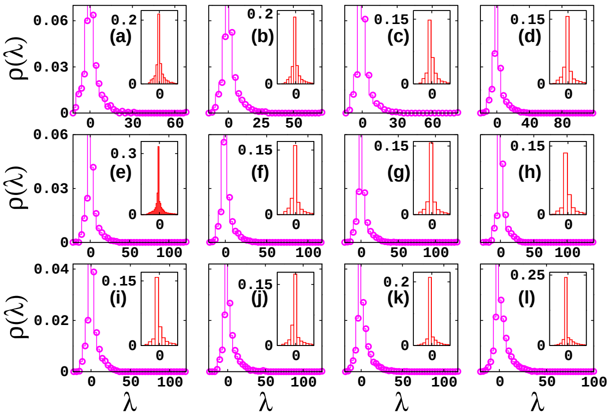

Figure S3: Spectral density distribution $\rho(\lambda)$ of Bollywood networks. [(a)-(l) stand for 1953-57, 1958-62, 1963-67, 1968-72, 1973-77, 1978-82, 1983-87, 1988-92, 1993-97, 1998-02, 2003-07 and 2008-12, respectively]. Inset depicts peak of distribution.

Eigenvalue plots of Bollywood datasets (Fig. S4) demonstrate the presence of few eigenvalues outside the bulk region. Datasets of 1913-27 do not exhibit formation of bulk due to scarcity in number of data points. Datasets of 1928-1952 depict separation of eigenvalues from bulk indicating existence of community structure (please refer main article for elaboration).

1.3.1 Nearest neighbor spacing distribution (NNSD)

Fig. S5 depicts NNSD of Bollywood networks. Discussion on NNSD is provided in the main article.

1.3.2 Δ_3 Statistics

It can be seen from Fig. S6 that the statistics agrees very well with the RMT prediction for some length for certain sets, and for some sets they do not follow RMT prediction of GOE statistics at all. The range for which $\Delta_3(L)$ statistics follows RMT prediction can be interpreted as providing measure of randomness in underlying network [16]. The length of the spectra which follow RMT prediction of GOE statistics is written in table 1 of main article. In some of the

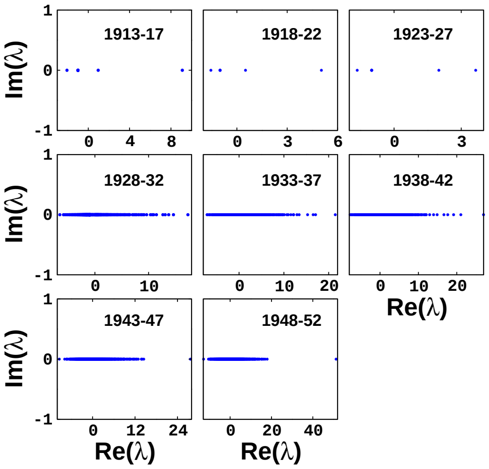

Figure S4: Separation of lone eigenvalues from bulk of Bollywood datasets spanning between 1913-1952.

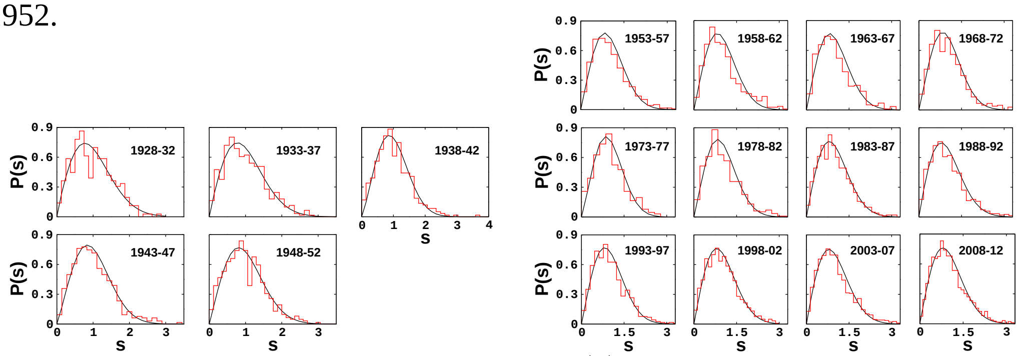

Figure S5: Nearest-neighbor spacing distribution $P(s)$ of the adjacency matrix of Bollywood networks. Histograms are numerical results and solid lines represent the NNSD of GOE.

sets namely 1953-57, 1958-62 and 1968-72 $\Delta_3(L)$ statistics does not follow RMT prediction at all.

1.4 Net payoff

Net payoff is a measure originally borrowed from management which is modified and used as a predictive means for assessing success. PageRank algorithm has also been used to assign ranks to nodes using a Markov chain based on the structure of the graph. This algorithm was used on Hollywood datasets to uncover the relative importance of a particular actor in the graph [6]. The

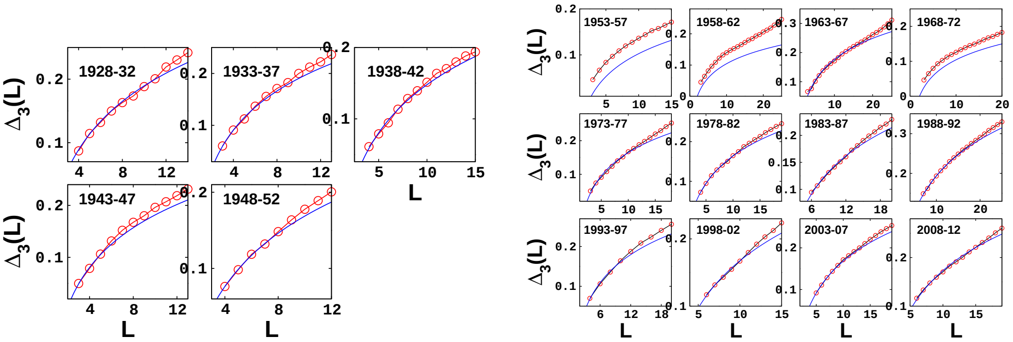

Figure S6: $\Delta_3(L)$ statistics of Bollywood networks. The solid line represents the GOE prediction, $\Delta_3(L)$ statistics follows the RMT prediction up to length L.

payoff defined here takes into account the essence of PageRank algorithm, alongwith other factors influencing the importance of a particular node. Statistics supporting the net payoff of lead male actors and female actors defined and discussed in the main article has been provided here in tables S2-S13. The 2003-07 span defies the trend of positive correlation between overlaps of the male actors appearing in top three consecutive positions of payoff list and their Filmfare nominations, where Amitabh Bachchan appears highest in the award nominees list. Here, it would be noteworthy to mention that the legendary Padma Shri (1984), Padma Bhushan (2001), Amitabh Bachchan (1969-2013), unlike all lead male actors of the yesteryear era, is the only one whose career never deteriorated. With 43 Filmfare nominations and being crowned as “Superstar of the Millennium” in 2000 at the Filmfare Awards, he redeems to be the superstar till date and is beyond all bounds.

Lead female actors appearing in top five positions of net payoff list have been observed to bag the top three positions in terms of Filmfare award nominations (manually selected) which is very precise in the recent dataset where top five of net payoff correspond to top four nominated lead female actors, except for Katrina Kaif, who does not have any Filmfare award nomination in 2003-2007 span still appearing at the 4th position in the top five (Table S8). She has been one of the most popular female actors in Bollywood since 2007, net payoff seems to be predictive

of her success.

Table S2: List of male actors holding top 10 positions in net payoff list of (a) and (b) datasets. Awards correspond to their award nominations in Filmfare in that particular span.

(a) 1953-1957

Actors	k	Award(s)
Ashok Kumar	115	-
Balraj Sahni	115	-
Raj Kapoor	78	1
Dilip Kumar	115	3
Shammi Kapoor	107	-
Dev Anand	84	-
Kishore Kumar	120	-
Ajit	113	-
Pradeep Kumar	114	-
Mahipal	85	-

(b) 1958-1962

List of Actors	k	Award(s)
Ashok Kumar	156	-
Dev Anand	115	3
Sunil Dutt	87	-
Dharmendra	61	-
Shammi Kapoor	114	-
Manoj Kumar	73	-
Rajendra Kumar	113	-
Shashi Kapoor	48	-
Pradeep Kumar	93	-
Kishore Kumar	97	-

Table S3: List of male actors holding top 10 positions in net payoff list of (a) and (b) datasets. Awards correspond to their award nominations in Filmfare in that particular span.

(a) 1963-67

List of Actors	k	Award(s)
Dharmendra	191	2
Ashok Kumar	160	3
Manoj Kumar	107	-
Biswajeet	115	-
Shashi Kapoor	103	-
Dev Anand	88	1
Sunil Dutt	110	2
Sanjeev Kumar	61	-
Dara Singh Randhawa	63	-
Rajendra Kumar	72	4

(b) 1968-72

List of Actors	k	Award(s)
Amitabh Bachchan	178	1
Sanjeev Kumar	247	2
Rajesh Khanna	234	5
Vinod Khanna	179	-
Shatrughan Sinha	204	1
Dharmendra	221	1
Jeetendra	204	-
Shashi Kapoor	115	-
Dara Singh Randhawa	156	-
Dev Anand	119	-

Table S4: List of male actors holding top 10 positions in net payoff list of (a) and (b) datasets. Awards correspond to their award nominations in Filmfare in that particular span.

(a) 1973-77

List of Actors	k	Award(s)
Rajesh Khanna	190	6
Sanjeev Kumar	234	5
Dharmendra	258	2
Amitabh Bachchan	299	4
Shashi Kapoor	195	2
Shatrughan Sinha	198	1
Vinod Khanna	191	2
Ashok Kumar	191	2
Vinod Mehra	178	-
Jeetendra	152	-

(b) 1978-82

List of Actors	k	Award(s)
Naseruddin Shah	117	3
Amitabh Bachchan	212	10
Dharmendra	181	-
Shashi Kapoor	226	-
Rajesh Khanna	184	3
Jeetendra	195	-
Raj Babbar	179	1
Sanjeev Kumar	221	5
Shatrughan Sinha	185	2
Om Puri	83	1

Table S5: List of male actors holding top 10 positions in net payoff list of (a) and (b) datasets. Awards correspond to their award nominations in Filmfare in that particular span.

(a) 1983-87

List of Actors	k	Award(s)
Naseruddin Shah	218	5
Javed Khan	198	-
Amitabh Bachchan	217	4
Dharmendra	199	1
Anil Kapoor	195	2
Om Puri	207	1
Suresh Oberoi	246	1
Mithun Chakraborty	233	-
Jackie Shroff	168	-
Raj Babbar	278	2

(b) 1988-92

List of Actors	k	Award(s)
Mithun Chakraborty	302	1
Jackie Shroff	220	1
Govinda	251	-
Anil Kapoor	225	3
Sanjay Dutt	249	1
Jeetendra	196	-
Rishi Kapoor	197	1
Dharmendra	269	-
Sunny Deol	155	1
Akshay Kumar	83	-

Table S6: List of male actors holding top 10 positions in net payoff list of (a) and (b) datasets. Awards correspond to their award nominations in Filmfare in that particular span.

(a) 1993-97

List of Actors	k	Award(s)
Shahrukh Khan	225	7
Raza Murad	296	-
Jackie Shroff	236	5
Sanjay Dutt	162	1
Kiran Kumar	324	1
Suniel Shetty	167	1
Naseruddin Shah	161	4
Govinda	186	4
Mithun Chakraborty	205	1
Akshay Kumar	235	1

(b) 1998-02

List of Actors	k	Award(s)
Shahrukh Khan	291	8
Jackie Shroff	398	2
Om Puri	286	3
Sanjay Dutt	304	2
Ajay Devgn	249	3
Salman Khan	199	4
Suniel Shetty	246	3
Govinda	208	7
Akshay Kumar	159	2
Mithun Chakraborty	173	-

Table S7: List of male actors holding top 10 positions in net payoff list of 2003-07 datasets. Awards correspond to their award nominations in Filmfare in that particular span.

List of Actors	k	Award(s)
Salman Khan	261	3
Irrfan Khan	201	1
Jackie Shroff	206	1
Ajay Devgn	228	6
Milind Gunaji	230	-
Akshay Kumar	326	4
Shahrukh Khan	246	9
Shakti Kapoor	315	-
Kay Kay Menon	216	1
Sanjay Dutt	322	4

Table S8: List of female actors in descending order of their net payoffs in 2003-07 span who are manually selected based on their popularity, Filmfare award nominations, income *www.filmfare.com*. Award(s) correspond to their award nominations in Filmfare in that particular span.

Name	Net payoff	Award(s)
Kareena Kapoor	0.49	4
Priyanka Chopra	0.46	4
Rani Mukerji	0.44	10
Katrina Kaif	0.39	-
Bipasha Basu	0.37	4

Table S9: List of female actors in descending order of their net payoff list of (a) and (b) datasets who are manually selected based on their popularity, Filmfare award nominations, income *www.filmfare.com*. Award(s) correspond to their award nominations in Filmfare in that particular span.

(a) 1998-02

Name	Net payoff	Award(s)
Sridevi	0.72	1
Rani Mukerji	0.58	2
Tabu	0.54	7
Mahima Choudhary	0.53	4
Aishwarya Rai	0.51	4

(b) 1993-97

Name	Net payoff	Award(s)
Manisha Koirala	0.51	5
Raveena Tandon	0.47	1
Tabu	0.43	3
Juhi Chawla	0.42	1
Madhuri Dixit	0.40	6

Table S10: List of female actors in descending order of their net payoff list of (a) and (b) datasets who are manually selected based on their popularity, Filmfare award nominations, income *www.filmfare.com*. Award(s) correspond to their award nominations in Filmfare in that particular span.

(a) 1988-92

Name	Net payoff	Award(s)
Madhuri Dixit	0.51	4
Juhi Chawla	0.43	2
Dimple Kapadia	0.33	1
Shilpa Shirodkar	0.29	-
Farha	0.28	-

(b) 1983-87

Name	Net payoff	Award(s)
Sadhana	0.56	-
Rekha	0.48	2
Meenakshi Seshadri	0.45	-
Hema Malini	0.45	-
Sridevi	0.44	1

Table S11: List of female actors in descending order of their net payoff list of (a) and (b) datasets who are manually selected based on their popularity, Filmfare award nominations, income *www.filmfare.com*. Award(s) correspond to their award nominations in Filmfare in that particular span.

(a) 1978-82

Name	Net payoff	Award(s)
Rekha	0.67	5
Sarika	0.63	1
Hema Malini	0.63	3
Parveen Babi	0.54	-
Shabana Azmi	0.54	2

(b) 1973-77

Name	Net payoff	Award(s)
Rekha	0.66	-
Hema Malini	0.62	6
Reena Roy	0.50	1
Parveen Babi	0.49	-
Zeenat Aman	0.46	1

Table S12: List of female actors in descending order of their net payoff list of (a) and (b) datasets who are manually selected based on their popularity, Filmfare award nominations, income *www.filmfare.com*. Award(s) correspond to their award nominations in Filmfare in that particular span.

(a) 1968-72

Name	Net payoff	Award(s)
Sulochana	0.78	-
Mumtaz	0.64	3
Hema Malini	0.60	-
Jaya Bachchan	0.48	2
Rekha	0.44	-

(b) 1963-67

Name	Net payoff	Award(s)
Sulochana	0.76	-
Mumtaz	0.74	-
Mala Sinha	0.48	3
Meena Kumari	0.38	6
Tanuja	0.38	-

Table S13: List of female actors in descending order of their net payoff list of (a) and (b) datasets who are manually selected based on their popularity, Filmfare award nominations, income *www.filmfare.com*. Award(s) correspond to their award nominations in Filmfare in that particular span.

(a) 1958-62

Name	Net payoff	Award(s)
Sulochana	0.55	-
Mala Sinha	0.50	1
Mumtaz	0.46	-
Meena Kumari	0.40	1
Vyjayantimala	0.38	2

(b) 1953-57

Name	Net payoff	Award(s)
Shyama	0.72	-
Meena Kumari	0.61	2
Sulochana	0.58	-
Vyjayantimala	0.51	1

References and Notes

[1] Newman M, E, J (2001) Scientific collaboration networks: I. Network construction and fundamental results. *Phys Rev E* 64: e016131(1-8).

[2] Watts D, J, Strogatz S, H (1998) Collective dynamics of ‘small-world networks. *Nature* 393: 440-442.

[3] Newman M, E, J, Strogatz S, H, Watts D, J (2001) Random graphs with arbitrary degree distributions and their applications. *Phys Rev E* 64: e026118(1-17).

[4] Neville J, Jensen D (2007) Relational Dependency Networks. *JMLR* 8: 653-692.

[5] Boldi P, Rosa M, Santini M, Vigna S (2011) Layered label propagation: A multiresolution coordinate-free ordering for compressing social networks. *Proc of the 20th international conference on World Wide Web*, (Hyderabad, India), pp 587-596.

[6] Boldi P, Rosa M, Vigna S (2011) Robustness of Social Networks: Comparative Results Based on Distance Distributions. *Soc Inform* 6984: 8-21.

[7] Albert R, Barabási A-L (2000) Topology of Evolving Networks: Local Events and Universality. *Phys Rev Lett* 85(24): 5234-5237.

[8] Albert R, Barabási A-L (2002) Statistical mechanics of complex networks. *Rev Mod Phys* 74(1): 47-97.

[9] Newman M, E, J (2003) The structure and function of complex networks. *SIAM Rev* 45(2): 167-256.

[10] Erdős P, Rényi A (1960) On the Evolution of Random Graphs. *Publications of the Mathematical Institute of the Hungarian Academy of Sciences* 5: 17-61.

[11] Barabási A-L, Albert R (1999) Emergence of scaling in random networks. *Science* 286: 509-512.

[12] Farkas I, J, Derényi I, Barabási A-L, Vicsek T (2001) Spectra of real-world graphs: Beyond the semicircle law. *Phys Rev E* 64: e026704(1-12).

[13] Dorogovstev S, N, Goltsev A, V, Mendes J, F, F, Samukhin A, N (2003) Spectra of complex networks. *Phys Rev E* 68: e046109(1-10).

[14] Mehta M, L (1991) *Random Matrices* (Academic Press, New York), 2nd Ed, pp 2-667.

[15] Brody T, A (1973) Statistical measure for repulsion of energy-levels. *Lett Nuovo Cimento* 7(12): 482-484.

[16] Jalan S, Bandyopadhyay J, N (2009) Randomness of random networks: A random matrix analysis. *Europhys Lett* 87: e48010(1-5).
